# Right lower lobectomy eight years after left pneumonectomy for a second primary lung cancer

**DOI:** 10.1186/1749-8090-8-46

**Published:** 2013-03-15

**Authors:** Yunpeng Liu, Peipeng Cui, Zhiguang Yang, Peng Zhang, Rui Guo, Guoguang Shao

**Affiliations:** 1 Department of Thoracic Surgery, First Hospital of Jilin University, Changchun, Jilin Province, 130021, PR of China; 2 Second Hospital of Jilin University, Changchun, Jilin, 130041, China; 3 Department of Thoracic Surgery, Siping Central Hospital, Siping, Jilin, 136000, China

**Keywords:** Second primary lung cancer, Pneumonectomy, Lobectomy

## Abstract

Lobectomy for second primary lung cancer in a patient with previous pneumonectomy is seldom done because most such patients either have inadequate pulmonary reserve or metastatic disease at other sites. This is different than when this type of surgery is done for benign disease where the lobe to be resected is already non functional. We report a case where successful right lower lobectomy for a second primary lung cancer was carried out in a 53 year old man who had had a left pneumonectomy eight years before. We conclude that, although this type of approach can be worthwhile, surgeons must be cautious and selective before doing so.

## Background

Contralateral lobectomy after pneumonectomy is rarely done for lung cancer [1-9] because most such patients have inadequate cardiopulmonary reserve or metastatic disease at distant sites. This is different than what is seen in benign lung disease where the lobe to be removed is already non functional and cardiopulmonary adjustments have occurred over the previous several years [10-12]. We are presenting a case in which a successful right lower lobectomy (RLL) for second primary lung cancer was performed eight years after left pneumonectomy.

## Case presentation

**Figure 1:**
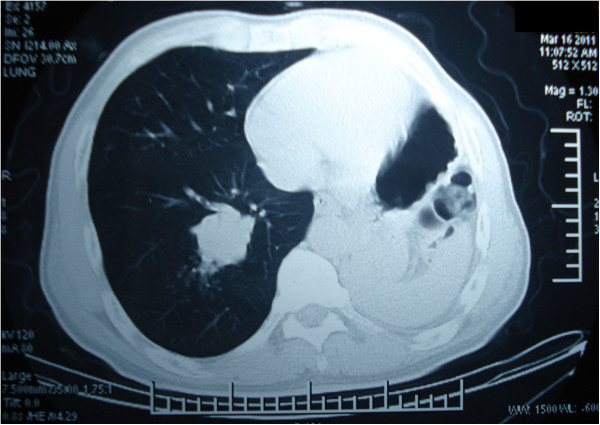
**Preoperative chest CT showing a 4 cm mass in the right lower lobe.** There is favorable hyperinflation of the right lung and normal postpneumonectomy status.

A 53 year old man with previous left pneumonectomy done eight years before for stage 2B squamous cell carcinoma was reassessed for increased cough and hemoptysis. The patient was otherwise well, had stopped smoking after his pneumonectomy, was not complaining of dyspnea, and had no significant other comorbidities. Chest CT showed a 4 cm mass in the RLL (Figure 1) without abnormal bronchopulmonary or mediastinal lymph nodes. At bronchoscopy, an endobronbchial tumor was seen in the medial basal segmental bronchus of the right lower lobe and biopsies confirmed the diagnosis of squamous carcinoma. Brain CT, isotopic bone scan, and abdominal ultrasonography were negative for distant metastases.

Preoperative pulmonary function studies showed moderate obstructive changes (Table 1) but what appeared to be adequate pulmonary reserve. Forced expiratory volume in the first second (FEV_1_) and carbon monoxide diffusing capacity (DLCO) were 43.6% (1,44 L) and 71.7% (20,19 ml/min/mmHg) of predicted values, respectivery. Cardiac ultrasonography showed that pulmonary artery pressure was normal at 22 mm Hg and the ejection fraction of the left ventricle was also normal. Based on this evaluation, we thought that the patient could tolerate a right lower lobectomy although our primary objective was to try to do a sublobar segmental resection.

**Table 1:** Pre and postoperative spirometric values

	**Preoperative**		**Postoperative (45 days)**	
	**Observed**	**% Predicted**	**Observed**	**% Predicted**
FEV1 (L)	1.44	43.6	1.40	42.2
FVC (L)	2.16	52.8	1.45	35.3
FEV1/FVC (%)	66.7	---	96.5	---
DLCO (Ml/min/mmHg)	20.19	71.7		

At operation, done through a standard posterolateral thoracotomy, it became obvious that a segmentectomy would not be technically possible and a right lower lobectomy with mediastinal lymphadenectomy was carried out. The resected tumor was pathologically staged T_2_N_2_ (pTNM, stage 3A) because micrometastases were found in one subcarinal (station 7) node on final pathological examination but the resection was felt to be complete (RO resection).

**Figure 2:**
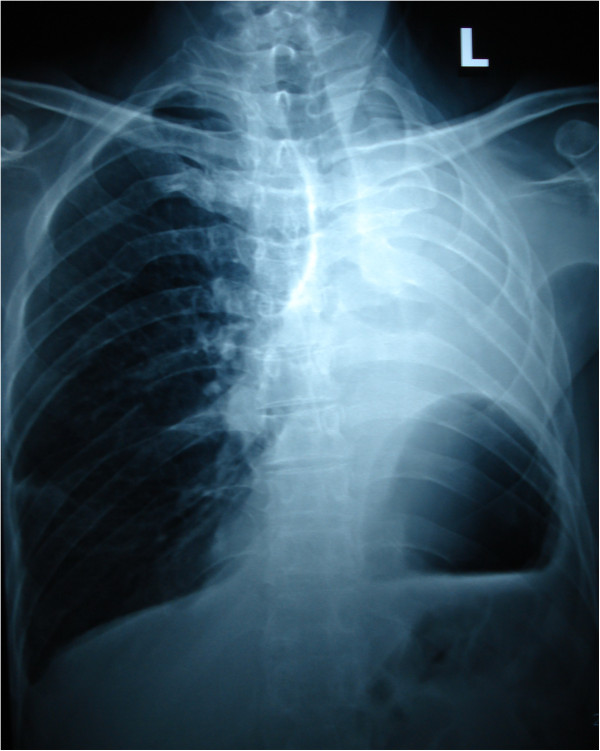
Standard radiograph done 25 days after surgery showing good expansion of the right upper and middle lobes and minimal postoperative inflammatory changes.

**Figure 3:**
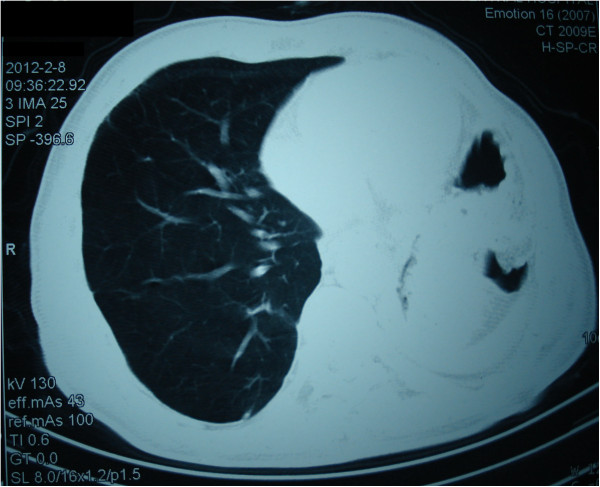
Chest CT done ten months after operation and showing good expansion of the residual lobes.

The patient had a normal postoperative course although he required oxygen supplementation for the first two weeks following the surgery. A standard chest radiograph (Figure 2) done when the patient was discharged from the hospital on postoperative day # 25 and a CT scan (Figure 3) done ten months after surgery show that the residual right upper and middle lobe are well expanded with minimal postoperative changes. Interestingly, pulmonary function studies done on the 45^th^ postoperative day (Table 1) show that FEV_1_ values are identical to those before the lobectomy.

### Discussion

Since pulmonary resection offers the best opportunity for long-term disease free survival in lung cancer patients, it is accepted that these patients should be offered a second operation should they develop a second primary after previous lobectomy. Such a decision is, however, more controversial, in patients with previous pneumonectomy expected to require lobectomy for complete resection of that second primary. Most previously reported cases are in the form of case reports (2, 3, 5, 8, 9) and in the four largest series ever published (1, 4, 6, 7), only two out of a total of 65 patients underwent a lobectomy while the remaining 63 had wedges or segmentectomies. This reluctance to do a lobectomy after previous pneumonectomy in the context of lung cancer is because patients are at high risk of operative mortality due to respiratory failure or pulmonary hypertension. This is different than what is seen in patients with benign diseases such as bronchiectasis or destroyed lungs which most of the time have been the result of repeated respiratory infections experienced in childhood [11]. In such cases, the lobe to be removed is nonfunctional and the cardiorespiratory system has had time to adjust to this situation over several years. One such reported patient lived an active life for more than three years with the RLL as his only lung tissue [10].

The most important issue in lung cancer patients expected to have subsequent lobectomy after previous pneumonectomy is how to select them for operation and how to predict which patients have enough cardiopulmonary reserve not only to survive the operation but also to have a good quality of life afterwards. Unfortunately, there is no easy formula to solve this dilemma and, although this type of surgery may occasionally be worthwhile like it was in our case, one has to be very cautious before making such a decision. Obviously, clinical history and spirometric assessment of pulmonary function are important and can be predictive of good outcome even if there are no magic numbers for FEV_1_, FVC, or DLCO that will accurately predict postoperative course and eventual quality of life. In our case, preoperative FEV_1_ was 1,44 L (43.6% of predicted) and, surprisingly, it remained identical at 45 days postoperatively. One can also do treadmill exercise-testing with measurement of maximal oxygen consumption (VO_2_max) and of arterial blood gases both at rest and during exercise. This was not done in our patient because he was in top physical condition having done manual work for all of his life.

Perhaps the most important preoperative assessment in that of cardiac function trying to predict if the patient will develop pulmonary hypertension during the postoperative period. Although direct measurements of pulmonary artery pressure can be done through heart catheterization, we think that a similar assessment can be achieved with cardiac ultrasonography. In our case, right ventricle and pulmonary artery pressures were normal as assessed by ultrasonography meaning that right heart function was normal and that the patient’s pulmonary arterial system could probably tolerate a lobectomy if required by intraoperative findings.

## Conclusion

This case indicates that lobectomy after previous pneumonectomy can be done in selected patients with adequate cardiopulmonary reserve. Since there are no preoperative values that are absolutely predictive of good outcome, surgeons must be very cautious before recommending operation.

## Consent

Written informed consent was obtained from the patient for publication of this case report and any accompanying images. Copy of the written consent is available for review.

## Competing interest

There is no potential competing interest with this article.

## Authors’ contributions

YL: main author wrote the paper. PC: performed the surgery and participated in the management. ZY: revised the manuscript. PZ and RG: participated in the design of the case report and performed the search in the literature. GS: performed the surgery reviewed the manuscript, and is the corresponding author. All authors read and approved the final manuscript.
